# Temporal Variation Dominates in Local Carabid Beetle Communities in Korean Mountains

**DOI:** 10.3390/insects12111019

**Published:** 2021-11-12

**Authors:** Yonghwan Park, Taewoong Jang, Jongkuk Kim, Su-Kyung Kim, Il-Kwon Kim, Chang-Jun Kim, Yasuoki Takami

**Affiliations:** 1 Forest Entomology and Pathology Division, National Institute of Forest Science, Seoul 02455, Korea; 2 College of Forest and Environmental Sciences, Kangwon National University, Chuncheon 24341, Korea; yogasin@nate.com (T.J.); jongkuk@kangwon.ac.kr (J.K.); 3 Division of Forest Biodiversity, Korea National Arboretum, Pocheon 11186, Korea; dorothyz@korea.kr (S.-K.K.); ilkwons91@korea.kr (I.-K.K.); changjunkim@korea.kr (C.-J.K.); 4 Graduate School of Human Development and Environment, Kobe University, Tsurukabuto 3-11, Nada, Kobe 657-8501, Japan; takami@people.kobe-u.ac.jp

**Keywords:** Baekdudaegan Mountain Range, biodiversity, carabidae, canonical correspondence analysis, community structure, environmental change

## Abstract

**Simple Summary:**

This study focused on spatial and temporal variation in carabid beetle communities and environmental factors, and the association between them at spatial and temporal scales, based on data collected over 5 years from nine study sites on three mountains. We found that carabid beetle communities exhibited significant temporal variation, and that the patterns of temporal variation differ between mountains. Temporal variation in communities was suggested to occur in response to variations in the local climate. Our results suggest that temporal surveys of communities and climates at local scales are important for predicting temporal changes in communities. Such investigations are expected to reveal an additional fraction of variation in communities, and to provide information on previously overlooked underlying processes, especially with respect to global community patterns and changes in wider spatial scales.

**Abstract:**

Spatial and temporal variation in ecological environments may result in spatial and temporal variation in communities. Temporal studies of biodiversity are essential for forecasting future changes in community structure and ecosystem function. Therefore, determining the mechanisms that drive temporal change in communities remains an important and interesting challenge in ecology. We quantified spatial and temporal variations in carabid beetle communities and site-specific environmental factors for 5 years at nine study sites on three mountains in the Baekdudaegan Mountain Range, Korea. Carabid beetle communities exhibited significant temporal variation, which was larger than spatial variations between and within mountains. Environmental factors mostly varied between sites within mountains. Community variation was only weakly associated with environmental factors at wide scales, i.e., between sites on three mountains, but was strongly associated at narrow spatial scales, i.e., between sites within one mountain. Our results indicate that temporal variation in communities occurs in response to variations in the local climate, and that the patterns of temporal variation differ between mountains. Thus, temporal surveys of insect communities and climates at local scales are important for predicting temporal changes in the communities.

## 1. Introduction

Spatial and temporal variation in ecological environments may reflect variability in spatial and temporal variation in communities [1]. Spatial factors, such as landscape, determine the presence of potential colonizers (species pool), their population dynamics, and their ability to reach particular patches in the landscape. Local environmental conditions determine the suitability of the habitat or patch for the organisms [2,3,4,5]. Life history traits and suitable habitat conditions vary across taxonomic groups and can influence species’ responses to ecological variables [6,7]. In contrast to intensive research into spatial variations in environments and communities [8,9], temporal variation in communities and its causes have received little attention [10], despite the fact that temporal studies of biodiversity are essential for forecasting future changes in community structure and ecosystem function. Thus, characterizing temporal change in communities remains an important and interesting challenge in ecology [11]. To understand the causes of spatial and temporal variation in communities, it is vital to quantify environmental variation and variation in community composition at temporal and spatial scales.

Carabid beetles are found in most terrestrial habitats, and their distribution often correlates with environmental variables [12,13]. These beetles are often selective of, or restricted to, a particular habitat [14,15,16] and are ideal organisms for assessing community responses to environmental change. Temporal changes in carabid beetle communities and the correlation between these changes and site-specific climatic factors were recently re-ported by the authors based on a 5-year survey of two mountains in Korea [17,18]. These two studies indicated that the patterns of temporal change (between years) in climatic conditions and in communities differed between sites within mountains, suggesting that both temporal and spatial (i.e., between sites on a mountain) variations contributed to the total variation in carabid beetle communities in these areas. Because carabid beetle fauna differs between mountains in this region [19], a larger spatial variation in communities is expected at wider spatial scales. However, the relative contributions of temporal and spatial variations to total variation in the carabid beetle community remains to be quantified.

The aims of this study were (1) to quantify the relative contribution of spatial and temporal variations in communities and environmental factors to total variation within an area, and (2) to examine the association between spatial and temporal variation in communities with environmental factors at different spatial and temporal scales. To this end, we examined two levels of spatial variation (between and within mountains) and one level of temporal variation (between years), to quantify and characterize the relative contribution of temporal and spatial variations to total variation in the beetle communities. We performed variance partitioning of measures of community composition and site-specific environmental factors, and examined covariation between them. We found large temporal variation in carabid communities but not in environments, and variation in community was not explained by variation in environmental factors at a wide geographical scale (i.e., between mountains). Based on these results, we suggest that site-specific temporal variation in beetle communities and environmental factors should receive more attention when predicting temporal changes in beetle communities.

## 2. Materials and Methods

### 2.1. Study Area and Field Survey

**Figure 1:**
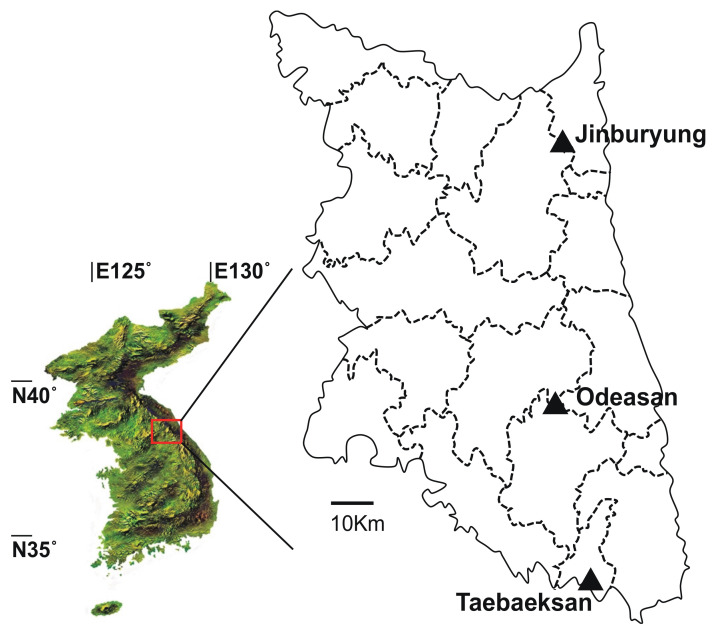
Locations of study sites on the Baekdudaegan Mountain Range, Korea.

Field surveys were conducted at nine sites, consisting of three sites on each of three mountains (Mts. Jinburyung, Odaesan, and Taebaeksan) in the Baekdudaegan Mountain Range, Korea. The mountains are separated from each other by 55–84 km. The Baekdudaegan Mountain Range is a watershed crestline 1400–1500 km long, running through most of the length of the Korean Peninsula, from Mt. Paektusan in the north to Mt. Jirisan in the south (Figure 1). Oak trees (*Quercus* spp.) provide the dominant cover at all study sites, with *Pinus densiflora* (Korean red pine), *Pseudostellaria palibiniana*, and *Dioscorea quinqueloba* present as understory vegetation. The study sites vary in litter layer development and understory vegetation (Table A1).

Changes in environmental variables at each study site were measured using HOBO data loggers (Onset Computer Corporation, Bourne, MA, USA). These loggers recorded air temperature, air humidity, light intensity, and soil temperature hourly from June 2010 to September 2015. Each logger was attached to a tree trunk 120 cm above the ground. Soil temperature was measured 5 cm underground. The data were downloaded directly from each logger every month. This system cannot measure precipitation; generally, this is difficult to measure hourly, although it is an important environmental parameter in predicting carabid beetle communities. Air humidity (relative humidity) was expected to be closely related to precipitation and was used as a surrogate.

To quantify community composition and its variation at each study site, carabid beetles were collected twice a year (July and August) for 5 consecutive years (2011–2015) using pitfall traps (plastic cups, 7.0 cm in diameter and 8.0 cm deep) containing an attractant (powder of silkworm pupae) [15,20]. July and August were chosen for sampling because adult carabid beetles are active during this period. For each sampling, 200 traps were set, in two or three lines, at 2 m intervals at each study site, and beetles were collected 24 h later. The exact position of the trap lines was changed arbitrarily each time when samples were taken from within the study sites to avoid effects of previous captures. All carabid beetles captured were counted and identified to the species level, except for three *Pterostichus*, three *Synuchus*, and one *Dolichus*; these species were regarded as morphospecies. The data of two samplings in a year were pooled, and constituted the sample for that site, month, and year, resulting in 45 data points across the nine sites over 5 years.

The sampling scheme (200 traps for 24 h) differs from that used in other studies [12,16], which used fewer traps for longer time periods (several weeks to months). Short time surveys can be influenced by transient changes in climate conditions and can result in larger sampling errors, such as dropout of rare species; however, long-term (but fewer traps) surveys can be influenced by spatial variations in microhabitats. These limitations were compensated for by using many traps with attractant and repeated samplings.

### 2.2. Environmental Factors

To summarize the environmental variables, we first calculated daily means from the data collected at hourly intervals at each site. Then, 12 environmental factors were calculated for each collection: summer mean air temperature, winter mean air temperature, summer maximum mean air temperature, winter highest mean air temperature, summer minimum mean air temperature, winter lowest mean air temperature, summer mean air humidity, winter mean air humidity, summer mean illumination (insolation), winter mean illumination, summer mean soil temperature, and winter mean soil temperature. Winter variables were calculated from the data collected in the December to February period before the first month of sampling. Summer variables were calculated from the data collected in the period from June to the date of the second sampling in the year. Summer and winter environments were mainly examined because, for carabid beetles in this region, summer conditions may be important for reproduction and population growth, and winter conditions may be important for survival during hibernation [21]. These environmental factors are associated with spatial and temporal variation in carabid beetle communities, as reported in our previous studies [17,18].

### 2.3. Analysis of Communities

To quantify spatial and temporal variation in community composition, Sørensen’s indices were calculated between three sites on one mountain, based on data pooled for 5 years (spatial β-diversity between sites within the mountain), and between sites on three mountains, based on data pooled for three sites and 5 years (spatial β-diversity between mountains). Sørensen’s indices from 5 years of data obtained from within each site (temporal β-diversity within site) and from each mountain (temporal β-diversity within a mountain) were also calculated, the latter of which was based on the data pooled for three sites on one mountain. In addition, Shannon’s diversity indices [22] for the data from each site and for each year were calculated (α-diversity within site and year), based on data pooled for 5 years at each site (α-diversity within site), and data pooled for three sites and for 5 years within one mountain (α-diversity within a mountain).

To summarize and visualize variation in community composition in relation to environmental factors, we performed canonical correspondence analysis (CCA) based on 45 data points of carabid beetle community composition and the 12 environmental factors. CCA models based on the data from a single mountain were reported in our previous studies [17,18], and these results are referred to in the Discussion section. Statistical significance of the effects of environmental factors was determined using a randomization test based on 999 pseudo-replications. This analysis was performed using the vegan function in the software package R 3.1.3 [23].

### 2.4. Variance Partitioning

To quantify the relative contributions of spatial and temporal variation to total variation in environmental factors and communities, we constructed generalized linear models (GLMs) with each of the 12 environmental factors and five measures of community composition (species richness, individual abundance, diversity index, and the scores of the first two CCA axes) as dependent variables, and year, mountain, and site (nested within mountain) as independent variables. Identity link and normal distribution were assumed for metric data, and log-link and Poisson distributions were used for count data. Variation partitioning [24,25] was used to quantify the proportion of variance explained by different spatial and temporal scales. Adjusted squared deviance, an equivalent to adjusted coefficient of variation, was calculated for year, mountain, and site [26]. Variation partitioning was performed using the rda function in vegan.

## 3. Results

### 3.1. Spatial and Temporal Variation in Environmental Factors

**Figure A1:**
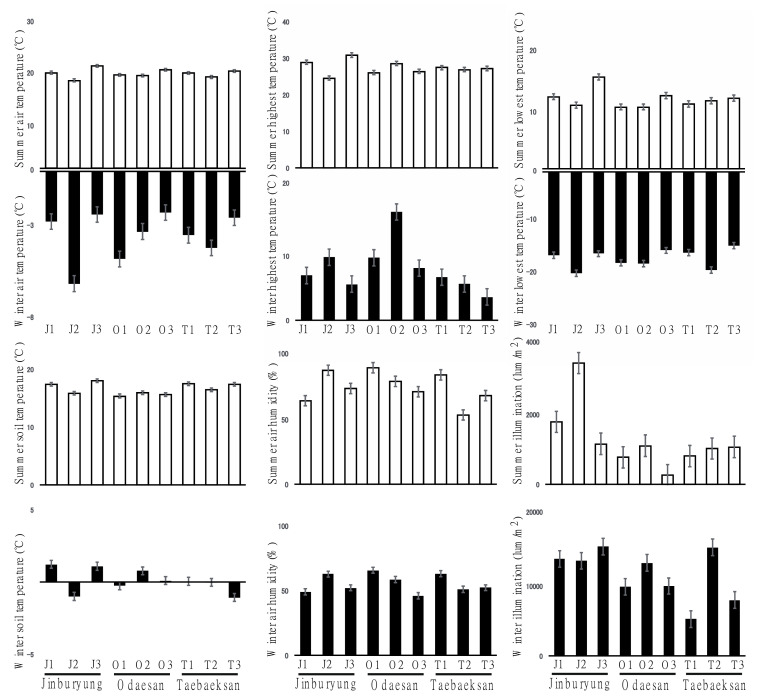
Temporal changes in environmental factors in the study sites on the Baekdudaegan Mountain Range, Korea. White and black bars refer to summer and winter environmental factors, respectively.

Environmental factors varied between mountains and between sites within a mountain. At all study sites during the study period, the monthly means of air temperature, soil temperature, and air humidity were highest in August and lowest in December or January (Figure A1).

Mountain, site, and year contributed to variations in environmental factors (Table 1 and Table A3). Summer mean soil temperature, summer mean illumination, and winter mean illumination varied significantly between mountains. All environmental factors except summer lowest mean air temperature differed significantly between sites. Summer mean air humidity, winter mean air temperature, and winter lowest mean air temperature varied significantly between years. Sites within a mountain accounted for the largest proportion of explained variability in 10 of the 12 environmental factors, whereas sites within a mountain accounted for the largest proportion of explained variability in summer mean soil temperature and summer mean illumination. Year accounted for the second largest proportion of explained variability in all environmental factors.

### 3.2. Spatial and Temporal Variation in Communities

We collected 7607 individual carabid beetles, belonging to 33 species in 13 genera (Table A2). At the genus level, *Synuchus* (2407 individuals), *Carabus* (2357), and *Pterostichus* (1567) were dominant, followed by *Pristosia* (555).

**Figure 2:**
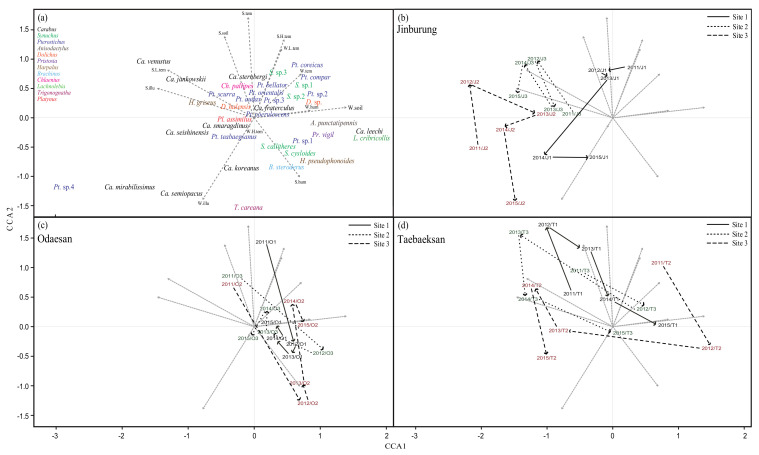
Canonical correspondence analysis (CCA) of temporal variation in communities. Arrows indicate changes in carabid beetle community composition in each of the 5 years of sampling: (**a**): each species, (**b**): carabid beetle communities in Jinburyung, (**c**): carabid beetle communities in Odaesan, (**d**): carabid beetle communities in Taebaeksan.

The 45 community data points were segregated along two CCA axes (Figure 2). Variations along the CCA1 axis explained 11.7% of total variation, and were associated with variations in summer mean air temperature, summer highest mean air temperature, winter mean illumination, and summer mean soil temperature. Variations along the CCA2 axis explained 7.5% of total variation, and were associated with variations in summer lowest mean air temperature, winter mean air humidity, summer mean illumination, and winter mean soil temperature (Figure 2a).

The patterns of temporal change in community composition differed between mountains (Figure 2b–d). Community compositions in Jinburyung and Odaesan varied mostly along the CCA2 axis. By contrast, community composition in Taebaeksan varied along both the CCA1 and CCA2 axes. Community composition tended to vary least in Odaesan, with the exception of the sample from 2011. Variation between the 5 years of replications in each site was larger than variation between sites, indicating that this analysis mainly captured changes between years within each site.

Diversity indices revealed that temporal variation in communities over 5 years was as large as spatial variation between sites within mountains and between mountains (Table 2): temporal β-diversity indices ranged from 0.32 to 0.58, whereas spatial β-diversity ranged from 0.40 to 0.53. α-diversity ranged from 0.36 to 1.31 in each site and year, and increased when pooled over 5 years (1.04–1.28). Spatial β-diversity within a mountain ranged from 0.293 to 0.486 in each mountain and year, and decreased when pooled over 5 years (0.239–0.281). By contrast, temporal β-diversity within a site did not change when pooled over 3 sites within a mountain.

Mountain, site, and year were responsible for the variation in the carabid communities (Table 3 and Table A4). Diversity index, species richness, individual abundance, and CCA1 were significantly explained by mountain, and individual abundance and CCA1 were significantly explained by sites within a mountain. All measures of community composition, except CCA2, were significantly explained by year. Year accounted for the largest proportion of explained variability in diversity, richness, and CCA2, and for the second largest proportion of explained variability in abundance. Mountain accounted for the largest proportion of explained variability in abundance and CCA1, and for the second largest proportion of explained variability in diversity and richness. Sites within a mountain accounted for the second largest proportion of explained variability in CCA1 and CCA2.

## 4. Discussion

We quantified spatial and temporal variation in carabid beetle communities and their background environments in the Baekdudaegan mountains in Korea over a 5-year continuous survey period. We found that temporal variation in communities was predominant and mostly larger than spatial variation between sites on a mountain and between mountains. For environmental factors, spatial variation between sites within a mountain represented the largest proportion of explained variation, followed by temporal variation, which accounted for the second largest proportion of explained variation. These results indicate that temporal change constitutes a significant component of variation in communities and environments but affects these components to different degrees. Because the geographical scale of this study was relatively narrow (Baekdudaegan Mountain Range in the Korean Peninsula), and the temporal scale was limited to 5 years, surveys performed at wider geographical and longer temporal scales may change the relative contributions of spatial and temporal variation, which may warrant further study.

Which factor (or factors) was responsible for the observed temporal variation in carabid beetle communities? Insufficient survey size can randomly bias the observed composition of a community (for example, as a result of random dropout of species) and may introduce apparent temporal fluctuation of communities. However, our results indicate this may not be the case. When community samples were pooled for 5 years within sites, α-diversity increased and spatial β-diversity across sites within a mountain decreased, whereas this tendency was not observed in temporal β-diversity (Table 2). Temporal β-diversity did not change after pooling for three sites within a mountain (Table 2). This inconsistency is not predicted by random dropout of species, and suggests that variation in observed community composition over years within a site was not random. Thus, insufficient survey size cannot be regarded as a major factor in the temporal variation in carabid beetle communities detected in this study. Alternatively, the results of CCA suggested that communities traced similar trajectories between sites within a mountain (Figure 2), which suggests that some deterministic factor played a role.

A change in the local environment may cause temporal variation in communities. Our previous studies indicated that spatial and temporal variation in carabid beetle communities occurred in response to changes in local environmental factors [17,18]. However, the results of the present study, based on surveys on three mountains, differ from those of our previous studies. The results for CCA showed no significant effect of environmental factors, and variance partitioning revealed that the relative contribution of temporal variation differed between environmental factors and community composition. A possible cause for this inconsistency is that the effects of environmental factors on communities differ between mountains. The results of CCA (Figure 2) revealed that temporal change in communities is similar between sites within a mountain, but different between mountains. These differences between mountains may indicate that the communities responded differently to local climate change, masking an overall tendency when analyzing the three mountains together. The response of local communities can be determined by local environmental factors, as suggested by the results of CCA obtained separately for each mountain: within Mt. Odaesan, summer mean air temperature, summer mean illumination, winter mean soil temperature, and winter mean illumination significantly explained community variation across three sites and 5 years [17]. Similarly, summer mean air humidity, winter mean illumination, and winter mean soil temperature also significantly explained community variation across three sites and 5 years on Mt. Jinburyung and Mt. Taebaeksan, respectively [18]. These results indicate that temporal changes in communities are determined by local environmental changes within a site and/or between sites within a mountain.

It is also possible that environmental factors not investigated in this study may influence carabid beetle communities. The phenology of carabid beetles can be related to their life cycles: spring breeder species reproduce in the spring and overwinter as adults, and the autumn breeder species reproduce in autumn and overwinter as larvae [27]. Thus, phenology in spring and autumn breeders may be influenced by changes in conditions during periods of temperature increase and decrease, respectively [28]. Further research to examine whether environmental variables in spring and autumn are associated with variation in communities is warranted.

Ecological processes other than responses to environmental factors may also result in temporal variation in communities. Ecological and reproductive interaction between species can influence the population dynamics of species. Resource competition between species exerts a negative effect by affecting access to resources [29,30]. Species can also interact negatively with each other through reproductive interference [31,32]. Carabid beetles have traditionally been regarded as an example of the lack of resource competition in animal communities [14,33], whereas reproductive interference is occasionally evident [34]. Further study is necessary to determine the effect of interspecific interaction on temporal change in carabid beetle communities in this study area.

Our results indicate that carabid beetle communities exhibit significant temporal variation. This highlights the importance of conducting temporal surveys of communities at local scales. Such investigations are expected to reveal an additional fraction of variation in communities, and to provide information on the underlying processes that have been overlooked, especially in studies of global community patterns and changes at wider spatial scales. Our results also suggest that detailed observation of local climates is necessary for predicting temporal change in communities. Such predictions may prove difficult if data are obtained at wider geographical scales. When responses of local communities to local environments differ between sites, statistical modeling of community responses to environmental factors may be difficult at wider geographical scales. This study calls for novel schemes for the statistical modeling of community changes that takes into account temporal variation.

## Figures and Tables

**Table 1:** Variation partitioning of environmental factors among explanatory variables.

	Mountain	Site within a Mountain	Year	Error
Summer mean air temperature	0.002	**0.512** ***	*0.* *094*	0.392
Summer highest mean air temperature	*0.* *054*	**0.644** ***	0.022	0.280
Summer lowest mean air temperature	0.078	**0.** **235**	*0.* *153*	0.534
Summer mean soil temperature	**0.188** *	0.145 *	*0.* *151*	0.516
Summer mean air humidity	0.084	**0.395** **	*0.219* *	0.302
Summer mean illumination	**0.216** **	*0.203* **	*0.203*	0.549
Winter mean air temperature	0.009	**0.536** ***	*0.* 274 *	0.182
Winter highest mean air temperature	0.038	**0.670** ***	*0.* *089*	0.203
Winter lowest mean air temperature	0.017	**0.347** *	*0.288* **	0.347
Winter mean soil temperature	0.062	**0.281** *	*0.* *188*	0.469
Winter mean air humidity	0.007	**0.457** **	*0.* *114*	0.422
Winter mean illumination	*0.264* **	**0.480** ***	0.062	0.194

The factors with the first and second largest effects are shown in bold and italics, respectively. *** *p* < 0.001, ** *p* < 0.01, * *p* < 0.05.

**Table 2:** Spatial and temporal variation in carabid beetle communities.

	Total (Pooled for 5 Years)	2011	2012	2013	2014	2015	Temporal *ß*
Jinburyung (pooled for three sites)	1.19	0.96	1.11	1.08	1.08	1.14	**0.367M**
J1	1.18	0.64	1.08	0.96	0.98	1.11	**0.327**
J2	1.04	0.81	0.77	0.93	0.85	0.96	**0.326**
J3	1.15	0.70	1.02	0.92	1.03	1.11	**0.393**
Spatial *ß* within Jinburyung	**0.239**	**0.318**	**0.352**	**0.340**	**0.293**	**0.327**	**-**
Odaesan (pooled for three sites)	1.28	1.13	1.15	1.20	1.20	1.31	**0.332**
O1	1.24	1.02	1.01	1.13	1.06	1.13	**0.327**
O2	1.26	1.07	0.92	1.11	1.18	1.25	**0.359**
O3	1.24	0.62	1.00	1.16	1.10	1.22	**0.324**
Spatial *ß* within Odaesan	**0.254**	**0.407**	**0.431**	**0.486**	**0.430**	**0.475**	**-**
Taebaeksan (pooled for three sites)	1.26	1.01	0.82	1.00	1.13	1.22	**0.573**
T1	1.20	0.87	0.36	0.86	1.11	1.07	**0.568**
T2	1.18	0.71	0.83	0.91	0.90	1.10	**0.575**
T3	1.19	0.65	0.76	0.77	0.96	1.17	**0.562**
Spatial β within Taebaeksan	**0.281**	**0.354**	**0.320**	**0.317**	**0.389**	**0.374**	-
Spatial β between mountains	**0.472**	**0.446**	**0.527**	**0.450**	**0.520**	**0.404**	-

Plain and bold numbers indicate α- and β-diversity indices, respectively.

**Table 3:** Variation partitioning of carabid community composition among explanatory variables.

	Mountain	Sites within a Mountain	Year	Error
Diversity	*0.264* **	0.051	**0.373** **	0.312
Richness	*0.204* ***	0.006	**0.562** ***	0.229
Abundance	**0.352** ***	0.062 *	*0.313* **	0.273
CCA1	**0.404** ***	*0.167* ***	0.048 ***	0.381
CCA2	0.110	*0.* *131*	**0.173**	0.586

Factors with the first and second largest effects are shown in bold and italics, respectively. *** *p* < 0.001, ** *p* < 0.01, * *p* < 0.05.

## Data Availability

Not applicable.

## Appendix A

**Table A1:** Habitat and environmental information on the study sites on the Baekdudaegan Mountain Range, Korea.

Site		Latitude Longitude	Slope	Altitude (m)	Gradient (°)	Dominant Tree Species	Understorey Vegetation Species
Jinbu-ryung	J1	38°16′00.31″ N 128°23′07.14″ E	NW	694	20	*Quercus mongolica*, *Tilia mandshurica*, *Synplocos chinensis*	*Synurus deltoides*, *Impatiens textori*, *Isodon excisus*
	J2	38°15′53.01″ N 128°20′01.94″ E	N	1038	10	*Quercus mongolica*, *Acer pseudosieboldianum*, *Tilia mandshurica*	*Carex siderosticta*, *Hepatica asiatica*, *Primula jesoana*
	J3	38°17′03.36″ N 128°21′40.76″ E	W	323	10	*Styrax obassia*, *Quercus mongolica*, *Pinus densiflora*	*Pseudostellaria palibiniana*, *Dioscorea quinqueloba*, *Isodon japonicus*
Odae-san	O1	38°44′31.86″ N 128°37′11.08″ E	W	741	25	*Quercus mongolica*, *Ulmus davidiana var. japonica*, *Fraxinus rhynchophylla*	*Brachybotrys paridiformis*, *Carex siderosticta*, *Lespsdeza japonica*
	O2	38°45′58.29″ N 128°36′20.22″ E	SW	1062	32	*Quercus mongolica*, *Tilia mandshurica*, *Acer pseudosieboldianum*	*Sasa borealis*, *Carex siderosticta*, *Viola albida*
	O3	38°47′10.76″ N 128°37′09.83″ E	N	668	20	*Fraxinus rhynchophylla*, *Pinus densiflora*, *Acer pseudosieboldianum*	*Disporums milacinum*, *Arisaema amurense*, *Isodon japonicus*
Tae-baek-san	T1	37° 7′22.90″ N 128°57′32.88″ E	W	968	28	*Betula davurica*, *Pinus densiflora*, *Quercus mongolica*	*Athyrium niponicum*, *Syneilesis palmata*, *Festuca ovina*
	T2	37°09′38.22″ N 128°53′10.35″ E	W	1274	36	*Quercus mongolica*, *Ulmus davidiana var. japonica*, *Fraxinus rhynchophylla*	*Astilbe koreana*, *Carex humilis*, *Viola albida*
	T3	37°06′36.02″ N 128°51′30.24″ E	SW	704	35	*Quercus mongolica*, *Fraxinus rhynchophylla*, *Tilia mandshurica*	*Sasa borealis*, *Tripterygium regelii*, *Carex siderosticta*

**Table A2:** Numbers of carabid beetles found on the Baekdudaegan Mountain Range, Korea, on three mountains, each sampled for two months per year over 5 years.

	Jinburyung					Odaesan					Taebaeksan					Total
Species	2011	2012	2013	2014	2015	2011	2012	2013	2014	2015	2011	2012	2013	2014	2015	
*Carabus (Eucarabus) sternbergi*	12	74	53	22	30	115	55	59	199	77	21	15	88	31	56	**905**
*Carabus (Leptocarabus) seishinensis*	69	31	41	62	53	25	29	34	61	41	22	1	41	87	63	**660**
*Carabus (Leptocarabus) semiopacus*	3	26	14	21	103		10	44	26	50			7	4	85	**393**
*Carabus (Leptocarabus) koreanus*				8										1		**9**
*Carabus (Morphocarabus) venustus*	1	27		26	22	4		7	8	3			13	13	2	**126**
*Carabus (Tomocarabus) fraterculus*	2			5	2	1		5	12	9	3	1		5	3	**48**
*Carabus (Coptolabrus) smaragdinus*	5	7	3	2	6	7	3	5	11	13			3			**65**
*Carabus (Coptolabrus) jankowskii*	1	8	12	3	6	3	9	5	4	7		1	25	35	17	**136**
*Carabus (Acoptolabrus) mirabilissimus*	7					1	1		1			1		1		**12**
*Carabus (Acoptolabrus) leechi*										1						**1**
*Synuchus callitheres*	4	9	19	1	57	23	56	74	100	97	2		10	19	86	**557**
*Synuchus cycloides*		3	3			10	53	112	66	82		4			101	**434**
*Synuchus nitidus*	29	31	41	23	59	4	65	83	259	115	22		29	50	88	**898**
*Synuchus* sp.1	44	2	1	7	10	31	22	5	67	38	31		12	10	5	**285**
*Synuchus* sp.2	3		2	5	4				95	39		3		14	10	**173**
*Synuchus* sp.3									44	14						**58**
*Platynus assimilis*	7		3		10	11	27	42	27	56	2		2		40	**227**
*Poecilus versicolor*	5	23	20	7	26	13	43	9	43	28		8		37	37	**299**
*Pterostichus orientalis*	2	12	7			5		41	30	40	7		7	11	18	**180**
*Pterostichus audax*	11	17	12	19	41	7	3	38	52	69	13		10	40	62	**394**
*Pterostichus scurra*	6	30	9	12	30	19	15	36	43	49	2		6	31	104	**392**
*Pterostichus compar*		14	11			8	31	5	31	29	4		10		66	**209**
*Pterostichus bellator*	1	10	8		6	27	19	33	31	42	3	6	7	8	25	**226**
*Pterostichus teabaegsanus*						1		10			1		1		4	**17**
*Pterostichus coreicus*						18										**18**
*Pterostichus* sp.1				5	5	1	14	6	16	20	2		2			**71**
*Pterostichus* sp.2						9	2	4	17	19						**51**
*Pterostichus* sp.3									4	5						**9**
*Dolichus halensis*		9		7	10	1	2	12	10	34	4			12	28	**129**
*Dolichus* sp.						10		6								**16**
*Pristosia vigil*	4	1	9	15	35	6	72	119	105	94	3	12	3	15	62	**547**
*Anisodactylus punctatipennis*						1		1		13					5	**20**
*Harpalus griseus*	1	1				3		2								**7**
*Brachinus stenoderus*							5	5								**10**
*Lachnolebia cribricollis*										3						**3**
*Chlaenius pallipes*						4		4			1					**9**
*Trigonognatha coreana*					1											**1**
Total	**217**	**335**	**268**	**250**	**516**	**368**	**536**	**806**	**1362**	**1087**	**143**	**52**	**276**	**424**	**967**	**7607**

**Table A3:** Generalized linear models (GLMs) of environmental factors.

	Mountain		Site within a Mountain		Year	
	*χ* ^2^_2_	*p*	*χ* ^2^_6_	*p*	*χ* ^2^_4_	*p*
Summer mean air temperature	0.032	0.968	4.756	0.0005	1.041	0.398
Summer highest mean air temperature	1.196	0.313	10.41	< 0.0001	0.224	0.923
Summer lowest mean air temperature	1.780	0.181	2.052	0.068	1.809	0.146
Summer mean soil temperature	4.862	0.013	2.250	0.046	1.776	0.153
Summer mean air humidity	1.935	0.157	4.142	0.001	2.769	0.039
Summer mean illumination	5.777	0.006	3.241	0.007	0.332	0.855
Winter mean air temperature	0.180	0.836	5.371	0.0002	3.773	0.011
Winter highest mean air temperature	0.835	0.441	10.93	<0.0001	0.975	0.432
Winter lowest mean air temperature	0.368	0.694	2.582	0.024	4.047	0.008
Winter mean soil temperature	1.394	0.259	2.348	0.038	2.317	0.074
Winter mean air humidity	0.157	0.855	3.901	0.002	1.280	0.294
Winter mean illumination	7.518	0.002	13.07	<0.0001	0.663	0.622

**Table A4:** Generalized linear models (GLMs) of carabid beetle communities.

	Mountain		Sites within a Mountain		Year	
	*χ* ^2^_2_	*p*	*χ* ^2^_6_	*p*	*χ* ^2^_4_	*p*
Diversity	5.207	0.010	1.443	0.213	5.379	0.001
Richness	9.408	0.0004	2.171	0.054	6.646	<0.0001
Abundance	9.506	0.0003	2.273	0.044	4.856	0.003
CCA1	16.950	<0.0001	7.526	<0.0001	0.315	0.866
CCA2	10.100	0.066	1.448	0.211	2.137	0.930
